# CT during celiac artery angiography for localization of clinically suspected small insulinomas

**DOI:** 10.1186/s40644-018-0155-7

**Published:** 2018-06-26

**Authors:** Feng Duan, Yan-hua Bai, Li Cui, Jie-yu Yan, Xiao-hui Li, Xiu-qi Wang

**Affiliations:** Department of Interventional Radiology, the General Hospital of Chinese People’s Liberation Army, Beijing, 100853 China

**Keywords:** Computed tomography∙ digital subtraction angiography ∙ celiac artery ∙insulinoma∙ interventional radiology

## Abstract

**Background:**

To identify location and number of insulinomas before operation is very important for improving the cure rate. The objective of the study was to assess performance of CT during celiac artery angiography for preoperative localization of clinically suspected small insulinomas (< 2 cm in diameter).

**Methods:**

From January 2013 to November 2016, 42 patients with hypoglycemic symptoms underwent celiac artery angiography, superior mesenteric artery angiography and CT during celiac artery angiography by a combined CT/digital subtraction angiography system, MIYABI Angio CT plus an Artiszeeceiling (SIEMENS, Germany). Patient group consisted of 13 males and 29 females, age 17–69 years (average, 45.4 ± 13.5 y). After diagnosis, all 42 patients were operated. Obtained images were retrospectively analyzed and compared with findings from post-operation pathology.

**Results:**

All interventional radiology procedures were performed successfully with no complications. Sensitivity of angiography alone for insulinoma was 76.1% (32/42), at combined CT/digital subtraction angiography, 4 more nodules were found (sensitivity, 85.7%, 36/42), while 6 false-negatives were observed (all false negative lesions were less than 2 cm). A total of 64 ml to 80 ml contrast media was used per patient.

**Conclusion:**

CT during celiac artery angiography is a sensitive diagnostic procedure for localizing insulinomas. Combined with angiography, it can prioritize the pancreatic region for exploration and guide a pancreatic resection.

**Trial registration:**

Ethical approval was obtained from the Hospital Research Ethics Committee. Informed consent was obtained from all patients included in the study.

Duan Feng, Bai Yan-hua and Cui Li are co-first authors.

## Background

Insulinoma is a rare tumor of the pancreas, with an estimated incidence of 0.4 per 100, 000 person/year [1]. To date, resection is the best treatment option. Therefore, preoperative localization of the tumor is very important. This information can help surgeons to make decisions on type and extent of the surgical resection [2]. However, small insulinomas (< 2 cm in diameter), in patients with clinical and biochemical evidence of endogenous hyper-insulinemia, are often difficult to localize in the pancreas. This is because there are still some false-positive and falls-negative diagnoses using non-invasive imaging modalities. CT during celiac artery angiography is a novel imaging modality and may enhance sensitivity for detection of small insulinomas. Therefore, we evaluated the benefit of CT during celiac artery angiography for localization of primary tumors in patients with clinically and biochemically suspected small insulinomas.

## Methods

This retrospective study was approved by the Hospital Ethics Committee. Informed consent was obtained from all patients included in the study.

### General information

From January 2013 to November 2016, 42 patients with hypoglycemic symptoms underwent CT during celiac artery angiography examinations. Patient group consisted of 13 males and 29 females, age between 17 and 69 (average of 45.4 ± 13.5), and blood sugar level varied between 0.81 and 3.05 mmol/L (average of 2.08 ± 0.67 mmol/L).

All examinations were performed using a MIYABI Angio-CT plus an Artiszee ceiling (SIEMENS, Germany), a combined CT/digital subtraction angiography (DSA) system. The two modalities were combined into a single system to provide both morphological and functional data from the same tumor in a single imaging session.

Firstly, celiac and superior mesenteric arteriography was performed via selective catheterization using a 4-F hepatic artery catheter (Terumo, Tokyo, Japan). Selective gastro-duodenal arteriography and splenic arteriography (if necessary) were performed using 4-F hepatic artery catheter as well. The total contrast media volume (Ultravist 370, Bayer, Germany) and injection rate were 20 ml and 4 ml/s for celiac and superior mesenteric arteriography, 12 ml and 3 ml/s for selective gastro-duodenal arteriography, and 16 ml and 4 ml/s for selective splenic arteriography. A total of 64 to 80 ml contrast media was used per patient.

Secondly, CT during celiac artery angiography was performed using the following scanning parameters: 16 detector rows, 5 mm section, pitch factor of 0.75, reconstruction interval of 1.5 mm, gantry rotation time of 0.6 s, tube voltage of 130 kV, and automatically determined tube mA. After infusion of 24 ml Ultravist-saline mixture (1:1) at 4 ml/s for 4 s, arterial-phase scanning was started. Venous-phase scanning started 12 – 15 s after perfusion of contrast medium began, and late-phase scanning was started 18 – 23 s after initiating contrast material infusion. The scanning time (approximately 4 – 6 s) varied depending on pancreas size. The total volume of non-diluted contrast medium for enhanced CT scan was 12 ml.

Angiographic procedures were performed by radiologists who had at least 10 years of experience with abdominal angiography. CT images were evaluated by two experienced radiologists. Statistical analyses were performed by the author (D.F.). A commercial statistical software package (SPSS for Windows, version 16.0; SPSS, Chicago, Ill) was used for data analysis. The t test was used to test differences between preoperative and postoperative blood glucose levels. *P* values< 0.05 were considered to indicate statistically significant difference.

## Results

**Fig. 1:**
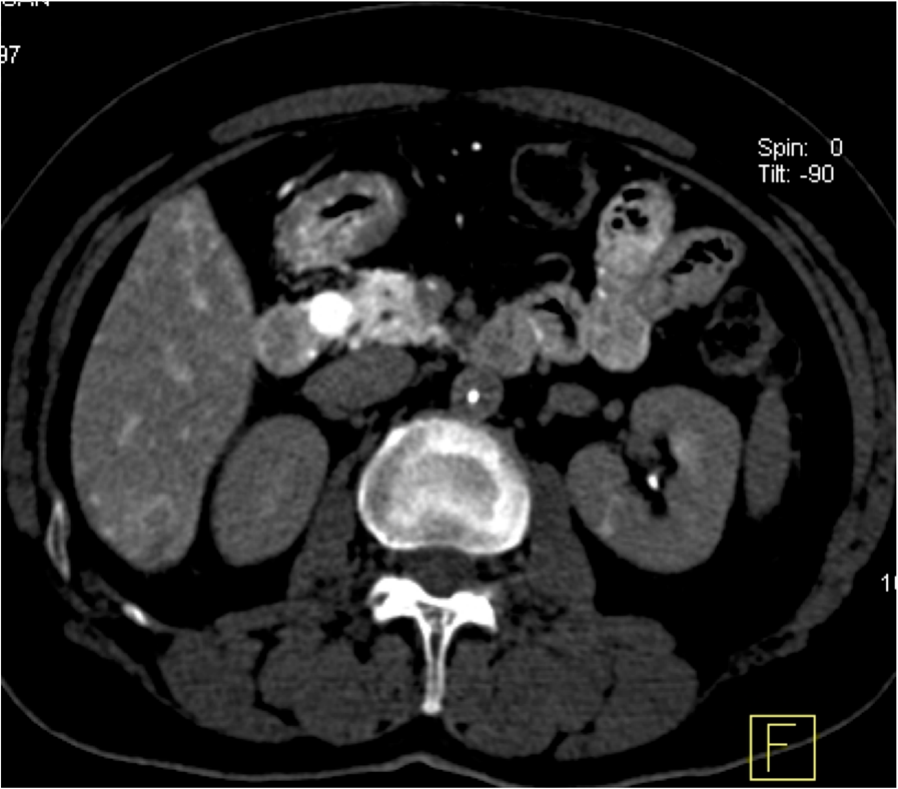
CT during celiac artery angiography shows an insulinoma in the pancreas head

**Fig. 2:**
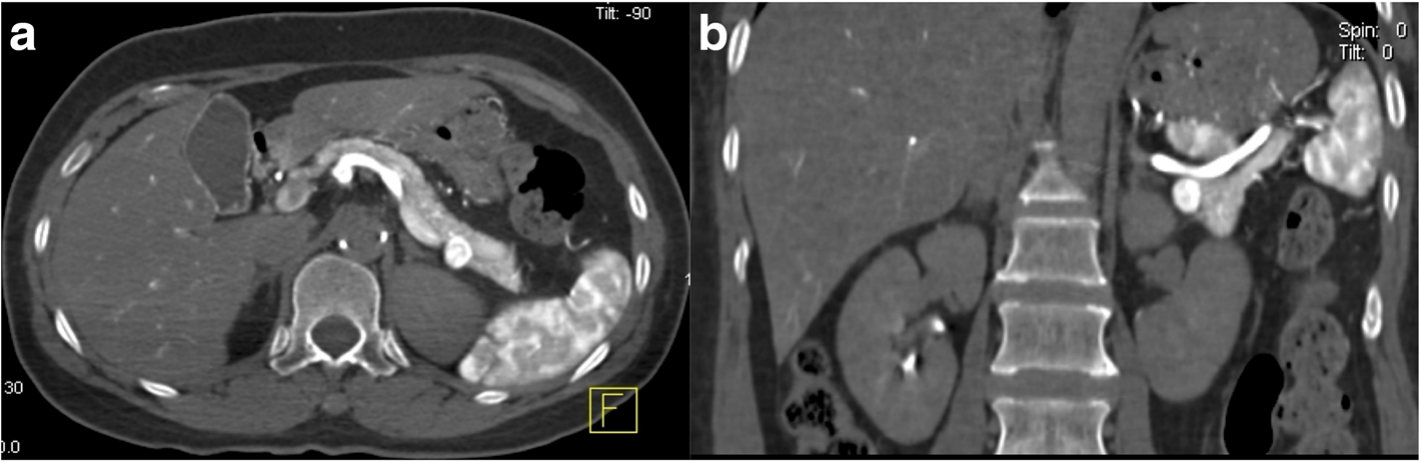
CT during celiac artery angiography shows an insulinoma in the pancreas tail. **a** Transverse CT view. **b** Coronal CT view

**Fig. 3:**
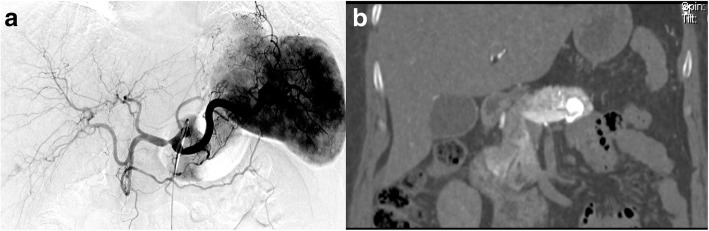
A 55-year old female. **a** Celiac artery angiography does not show any lesion. **b** CT during celiac artery angiography indicates a suspected lesion in the pancreas tail. Lesion is considered to overlay with spleen

All interventional radiology procedures were performed successfully with no complications. Subsequently, all 42 patients were operated, and in total 47 tumor nodules were collected. Most of the tumor nodules were grayish white, and only a few were grayish red or cherry red. All tumors ranged between a diameter of 0.5–3.5 cm (mean, 1.56 ± 0.8 cm), there were 3 lesions larger than 2 cm (2.5 cm, 2.5 cm and 3.5 cm, respectively), and the rest were less than 2 cm. All 3 patients with lesions larger than 2 cm were with multiple lesions. Excised lesions were of medium hardness, and of which 6 were from the head (Fig. 1), 10 from the body, 14 from the tail (Fig. 2a, b), 9 from the neck, and 8 from the uncinate process of the pancreas, with a multiple tumor rate of 9.5% (4/42 cases). Based on pathology results, all were benign insulinomas. Angiography imaging was consistent with surgery in 76.1% (32/42) of the cases (Fig. 3a), combined with angiography images, 4 more nodules were found in CT images, and CT imaging was consistent with surgery in 85.7% (36/42) of the cases (Fig. 3b), while 6 false-negatives were observed (all false negative lesions were less than 2 cm). A total of 64 to 80 ml contrast media was used per patient.

DSA manifestation of small insulinomas was characterized by homogeneous staining of the tumor, clear borders of majority of the tumors, enlargement of feeding arteries and increased tumor vessel density. Plain CT scans showed no significant differentiation or local elevated outline, and tumor density was not different from normal pancreas. Contrast enhanced scans indicated tumor nodules with clear borders, which present washout of contrast.

The hypoglycemic symptoms of all patients improved after operation. Blood glucose levels before operation were between 0.81 and 3.05 mmol/L (average of 2.08 ± 0.67 mmol/L), while blood glucose levels after operation were between 4.76 and 11.2 mmol/L (average of 7.30 ± 2.21 mmol/L), p<0.01.

## Discussion

Insulinomas are classified as neuroendocrine tumors derived of beta cells in the pancreas, which secrete insulin, according to the 2010 WHO, 2007 ENETS and 2010 UICC-pTNM (NETs) classification [3]. Most insulinomas are functional, with typical symptoms according to the Whipple triad. During a typical hypoglycemia episode, blood glucose decreases to < 2.8 mmol/l, which is quickly relieved after intake of glucose. Although clinical symptoms are evident, insulinomas are often small, with more than 80% of the insulinomas smaller than 2 cm in diameter [4]. In this group of patients, 92.9% of the insulinomas are < 2 cm. Because of their small size, they are likely to be missed during conventional imaging exams.

Common detection methods for insulinomas currently include non-invasive imaging modalities, such as ultrasonography (US), computed tomography (CT), magnetic resonance imaging (MRI) and 18-Fluoro-DOPA PET/CT scanning, and invasive pre-operative diagnostic procedures, such as endoscopic ultrasound (EUS), DSA, intra-arterial calcium stimulation test (ASVS), and trans-hepatic peri-pancreatic venous blood sampling (TPVB). There are pros and cons for each method, for example, the sensitivity of non-invasive preoperative imaging, for localization of lesions, is between 73.08 and 90% [5–8]. Conventional enhanced images sometimes are not able to accurately capture the most prominent phase of enhancement of a particular lesion, which makes it possible to get an equivocal results at conventional imaging [9]. Especially for small lesions, non-invasive imaging is not sufficient for preoperative localization of insulinomas. In case of the various invasive exam methods, endoscopic ultrasound is highly sensitive for diagnosis. However, findings can be sometimes false positive. Quality of findings obtained by endoscopic ultrasound imaging depends to a large extent on the experience of the examiner. Furthermore, some insulinomas are missed by preoperative EUS, because they are completely isoechoic [10, 11]. Conventional angiography DSA has low resolution. Especially lesions, which overlay with duodenum or spleen, can hardly be distinguished. It has been reported that the diagnosis accuracy of selective DSA on pancreas is 72–83.3% [12]. ASVS can only provide information on regional location of insulinomas, but cannot confirm size or exact position (for instance on pancreas surface or not). Importantly, insufficient preoperative imaging may affect the choice of operational methods in a negative way (e. g., if a minimally invasive laparoscopic enucleation is applicable) [13].

The combination of angiography with CT during celiac artery angiography showed several advantages: 1) it can provide greater enhancement of pancreatic tumors by the administration of contrast material directly into the proper artery. 2) CT provides significantly higher spatial resolution than DSA, thus it provides more precise diagnosis for small lesions, especially lesions, which overlay with duodenum or spleen. 3) Only local injection of contrast medium is required, so the dosage of contrast medium is greatly reduced compared to conventional CT. In our study, the dose of contrast medium for CT was 12 ml, and even with the addition of contrast medium for angiography, the total dose of contrast media was still lower than applied with a conventional CT. 4) Two exams can be performed simultaneously, and through integration of the two exam findings small insulinomas can more precisely be located. In particular, for iso-attenuating insulinomas, adjuvant observation of blood flow in the suspected lesions can help with identification of tumor regions [14]. Although current DSA includes dyna-CT function, its resolution is still lower than conventional CT. Moreover, the reconstruction region of dyna-CT is relatively small [15]; in some cases not the entire pancreas can be included. Consequently small insulinomas can likely be missed.

Limitations of the modality presented here include: 1, the study did not carry out a comparison of CT during celiac artery angiography and other “conventional” imaging modalities. Because the study was retrospective, the preoperative imaging algorithm was not standardized; the number of cases for each examination is too small to carry out comparative study. As the number of cases increases, we will conduct a comparative study with the conventional imaging modality; 2, CT during celiac artery angiography is an invasive exam method, thus it has a higher increased risk than noninvasive exams; 3, this exam still relies on the difference between tumorous arterial blood supply and normal tissue blood supply to identify tumor lesions. For that reason false negatives can incur when tumors have normal arterial blood supply. In this case, ASVS can be combined to increase the sensitivity.

## Conclusion

Together we conclude that CT during celiac artery angiography is a sensitive diagnostic procedure for localization of insulinomas. In combination with angiography, it can cover the pancreatic region for a prioritized exploration and guide pancreatic resections.

## Abbreviations

ASVS: intra-arterial calcium stimulation test
CT: Computed Tomography
DSA: digital subtraction angiography
EUS: endoscopic ultrasound
MRI: magnetic resonance imaging
TPVB: trans-hepatic peri-pancreatic venous blood sampling
US: ultrasonography
